# Tumor Lysis Syndrome in Patients With Solid Tumors: A Systematic Review of Reported Cases

**DOI:** 10.7759/cureus.30652

**Published:** 2022-10-25

**Authors:** Riyadh M Alqurashi, Husam H Tamim, Ziyad D Alsubhi, Alyazid A Alzahrani, Emad Tashkandi

**Affiliations:** 1 College of Medicine, Umm Al-Qura University, Makkah, SAU; 2 Medical Oncology, King Abdullah Medical City, Makkah, SAU

**Keywords:** allopurinol, immunotherapy, radiotherapy, chemotherapy, tumor lysis syndrome, solid tumors, systematic review

## Abstract

Tumor lysis syndrome (TLS) in patients with solid tumors is a rare and potentially fatal condition associated with anti-cancer treatment. Its outcome depends on awareness, identification of high-risk patients, and implementation of appropriate preventive measures. A systematic review was conducted according to PRISMA guidelines of case reports describing the occurrence of TLS in patients with solid tumors, primarily to identify potentially unrecognized or unusual clinical findings and outcomes. We searched the PubMed, EMBASE, and Cochrane databases and conference abstracts and performed manual searches for case reports and case series published in English and describing patients who developed TLS.

A total of 124 studies (118 case reports and six case series) describing the findings for 132 patients were included. The most common cancers were hepatocellular carcinoma (17%, n = 22), lung cancer (13%, n = 17), and melanoma (10%, n = 13). The most common risk factor was metastatic disease (75%, n = 100). TLS was induced by chemotherapy in 48% (n = 64) of the patients. Clinical manifestations of TLS developed within three days of anti-cancer treatment in 37% of the patients (n = 49), while 52% (n = 68) received the full dose of anti-cancer treatment. Gastrointestinal symptoms occurred in 33% of the patients (n = 44), hyperuricemia in 95% (n = 125), and elevated creatinine level occurred in 85% of the patients (n = 112), While 58% (n = 77) of the patients received intravenous fluids, only 49% received allopurinol, and 24% (n = 32) received rasburicase. A total of 101 patients (77%) were treated in the ward, and 54% (n = 71) died. The mortality rate associated with TLS in patients with solid tumors remains high. Adequate management requires awareness, early recognition, and identification of patients at high risk. Interdisciplinary team management is essential to reduce mortality.

## Introduction and background

Tumor lysis syndrome (TLS) is an oncological emergency that occurs secondary to the breakdown of intracellular components such as potassium, phosphorus, and nucleic acids [1]. The release of these products into the bloodstream leads to hyperkalemia, hyperphosphatemia, hyperuricemia, and hypocalcemia, inducing severe complications such as acute renal failure, cardiac arrhythmia, heart failure, seizure, and ultimately death if the patient is not managed appropriately [2,3]. Although the rapid destruction of malignant cells occurs after exposure to anti-cancer treatments such as chemotherapy, radiotherapy, monoclonal antibody treatment, radiofrequency ablation (RFA), corticosteroid treatment, hormonal therapy, and surgery, it can also occur in the absence of anti-cancer treatments, especially if the tumor is bulky or rapidly proliferating. These cases are categorized as spontaneous TLS [4-6].

TLS is commonly observed in hematological malignancies such as Burkitt or non-Burkitt lymphoma and acute leukemia. However, since solid tumors have a relatively prolonged doubling time and slower growth rate, and the effect of therapy takes longer time than hematological malignancies, TLS is rarely observed in solid tumors. However, some cases of TLS have been reported in patients with small-cell lung cancer, breast cancer, medulloblastoma, melanoma, and sarcoma. [7-13] The risk factors for TLS could be due to patient-related factors such as dehydration, chronic renal failure, elevated pretreatment lactate dehydrogenase (LDH) or uric acid levels, and azotemia or tumor-related factors such as bulkiness, rapid growth, or a tendency to spread to other organs, specifically the bone marrow [14]. TLS is an oncological emergency that needs to be recognized urgently, and if treated early, complications can be prevented, thereby improving the outcomes [15]. The Cairo-Bishop laboratory and clinical criteria are used to diagnose TLS (Table 1) [16]. The presence of two or more laboratory abnormalities starting either three days before or seven days after treatment of the tumor can be used to define laboratory TLS. However, clinical TLS is characterized by the appearance of two laboratory abnormalities and one or more clinical symptoms [17,18].

**Table 1:** Cairo-Bishop criteria for tumor lysis syndrome

Laboratory criteria	(≥2 of the following): Uric acid ≥ 476 μmol/mL (8 mg/dL) or 25% increase from baseline; Phosphorus ≥ 1.45 mmol/L (4.5 mg/dL) or 25% increase from baseline; Potassium ≥ 6.0 mmol/L (6 mEq/L) or 25% increase from baseline; Calcium ≤ 1·75 mmol/L or 25% decrease from baseline
Clinical criteria	Any of following with laboratory criteria: Creatinine ≥ 1.5 upper limit of normal. Cardiac arrhythmia or sudden death. Seizures.

TLS is a potentially fatal condition in patients with solid tumors and is associated with worse outcomes if it occurs spontaneously [16], It has a poor prognosis, especially if it is not diagnosed early; therefore, awareness, recognition, prevention, and early intervention are warranted to prevent the fatal consequences of TLS.

In this paper, we present a systematic review of the reported cases of TLS in patients with solid tumors that developed spontaneously or as adverse effects of anti-cancer treatments such as chemotherapy, immunotherapy, targeted therapy, and hormonal therapy. By describing the occurrence of TLS in patients with solid tumors, we primarily aim to identify potentially unrecognized or unusual clinical findings and outcomes. Also, determine the most common clinical manifestations, time to TLS, number of doses administered before TLS, treatment dosage used, presenting symptoms, and laboratory abnormalities. We also reported the management and clinical outcomes to identify patterns that could facilitate early diagnosis and management of this potentially fatal condition. 

## Review

Materials and methods

Search Method 

Digital databases were used including PubMed, EMBASE, and Cochrane from 1983 to July 1, 2020, for case reports and case series of TLS in patients with solid tumors. In addition, abstracts and presentations from relevant conference proceedings, including the American Society of Clinical Oncology (ASCO) and the European Society for Medical Oncology (ESMO) have been used. 

Study Selection and Eligibility criteria 

Two independent reviewers (ZA and HT) initially screened the abstracts and titles. Then, two other reviewers (AA and RA) assessed the full texts of the retrieved articles and resolved disagreements in conjunction with a third reviewer (HT). The eligibility criteria were as follows: case reports published in English, describing adults with solid tumors, and reporting spontaneous TLS or TLS that developed after anti-cancer treatments such as chemotherapy, targeted therapy, hormonal therapy, immunotherapy, or radiotherapy. We excluded studies involving hematological tumors, pediatric patients, and non-case reports/series. Keywords for the literature search included published case reports, case series, TLS, solid tumors, and anti-cancer treatment. The search strategy is provided in Appendix 1.

Data Extraction

This study was performed in accordance with the Preferred Reporting Items for Systematic Reviews and Meta-analyses (PRISMA) statement [19]. A protocol was created in advance, and data extraction for reported cases of spontaneous TLS or TLS that developed as an adverse effect of anti-cancer treatment was performed independently by two reviewers (ZA and AA), with disagreements resolved by a third reviewer (RA). 

We extracted data on patient characteristics (first author, year of publication, age, sex, type of cancer), risk factors (metastasis, elevated pre-treatment LDH level, bulky tumor, and pre-existing renal compromise), and comorbidities. The anti-cancer treatments administered in the cases included chemotherapy, immunotherapy, targeted therapy, hormonal therapy, and radiotherapy. The most common clinical parameters were time to TLS (1-2 days, ≥3 days, spontaneous), number of doses administered before TLS (1 dose, 2-3 doses, >3 doses, spontaneous), dosage of treatment used (full or reduced dose), presenting symptoms, and laboratory abnormalities (uric acid, phosphorus, potassium, calcium, creatinine, urea, and LDH levels). Lastly, we collected information regarding management and clinical outcomes, use of anti-TLS measures, location of treatment received (ward or ICU), and outcome (dead or alive).

Quality Assessment

We assessed the quality of each study by using the criteria recommended by the International Society for Pharmacoepidemiology (ISPE) and the International Society of Pharmacovigilance [20]. Two independent reviewers (HT and RA) assessed the quality of the included studies across the following domains: (i) relevance of the title for TLS, (ii) adequate description of clinical characteristics (demographics, medical history, physical examination, and outcomes (alive or dead)), (iii) adequate description of anti-cancer drugs (identification of the drug class, dosage, drug reaction, and concomitant therapy) and time to develop adverse events; (iv) adequate description of the adverse event (TLS); and (v) discussion section supporting the relationship between the anti-cancer drug and the reported adverse events (TLS). Each aspect was classified as yes, partial, or no. Any disagreements were resolved by a third reviewer. The results of the assessment are presented in Appendix 2.

Data Synthesis and Analysis

All data were analyzed using IBM SPSS Statistics for Windows, Version 25.0 (Released 2017; IBM Corp., Armonk, New York, United States). Descriptive statistics (mean, percentage, and standard deviation) were used to report continuous variables, and frequencies and percentages were used to present categorical variables.

Results

Study Characteristics

**Figure 1:**
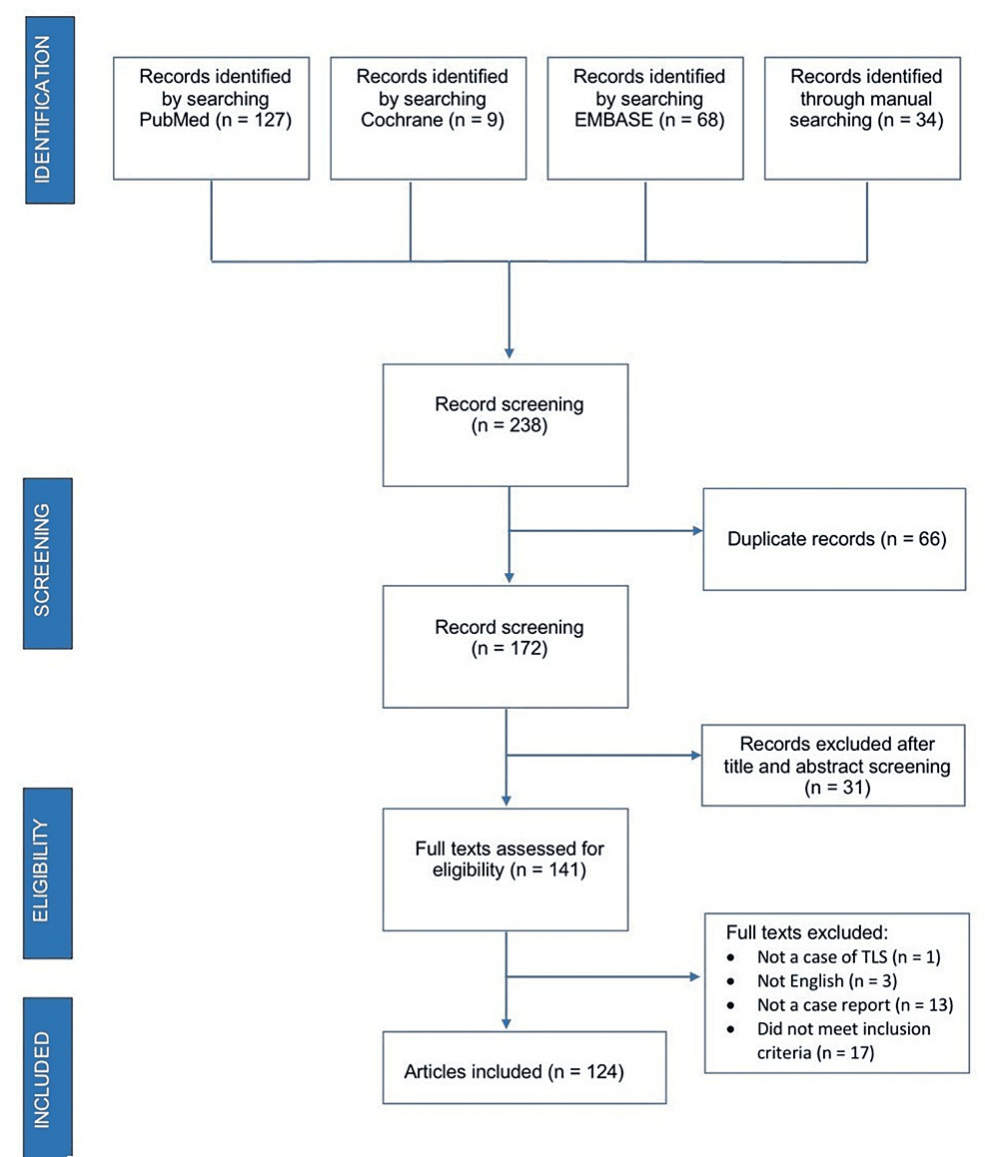
PRISMA flowchart of our study selection PRISMA: preferred reporting items for systematic reviews and meta-analysis; TLS: tumor lysis syndrome

In total, 238 citations were retrieved. After the removal of duplicates, we identified 172 relevant citations and reviewed the full publications. We excluded 17 studies since they were not case reports. We included 124 studies reporting on 132 patients as provided in Figure 1. The characteristics of the included studies are given in Appendix 3.

Quality Appraisal

The quality of the included studies was moderate to high since all included studies had relevant titles, adequate descriptions of patients’ demographic data (96.7%), current health status (95.1%), medical history (87.9%), physical examination findings (97.5%), and disposition (98%). The anti-cancer drugs were identified for all reported cases of drug-induced TLS, but the drug dosage was not provided in approximately one-quarter of the cases. The duration of drug administration, route, and first dose were reported (70.9%). Furthermore, concomitant therapy had no potential influence (94.3%). A description of the adverse event and severity was reported (92.7%), and an appropriate discussion supporting a causal link between the drug and the adverse events was provided (92.7%).

Patient Characteristics

The median age was 58 years (Interquartile range (IQR) 19-94 years) and the proportion of males was 62% (n = 83). The most common tumors were hepatocellular carcinomas (17%, n = 22), lung cancer (13%, n = 17), melanoma (10%, n = 13), breast cancer (10%, n = 13), prostate cancer (8%, n = 10), and colon cancer (8%, n = 11). The risk factors were metastatic disease in 75% of the patients (n = 100), elevated pre-treatment LDH level in 26% (n = 35), and bulky tumors in 25% (n = 33). The main comorbidities were hypertension, hepatitis B, and diabetes mellitus in 11%, 8%, and 6% of patients, respectively (Table 2).

**Table 2:** Characteristics of patients in the reported cases HCC: hepatocellular carcinoma; LDH: lactate dehydrogenase; HTN: hypertension; DM: diabetes mellitus; COPD: chronic obstructive pulmonary disease; CKD: chronic kidney disease ^A^ Other tumors included choriocarcinoma, osteosarcoma, oligodendroglioma, neuroendocrine tumors, Merkel cell carcinoma, vulvar tumor, gastrointestinal stromal tumors, pheochromocytoma, thymoma, and retroperitoneal soft tissue sarcoma. ^B^ Other comorbidities included congestive heart failure, cirrhosis, and arthritis

Patient characteristics	N (%)
Median age	58, (range 19-94) years
Sex	
Male	83 (62%)
Female	49 (37%)
Cancers	
HCC	22 (17%)
Lung cancer	17 (13%)
Melanoma	13 (10%)
Breast cancer	13 (10%)
Colon cancer	11 (8%)
Prostatic cancer	10 (8%)
Renal cell carcinoma	6 (5%)
Gastric cancer	6 (5%)
Ovarian cancer	5 (4%)
Uterine cancer	5 (4%)
Germ cell tumors	3 (2%)
Other^a^	21 (16%)
Risk factors	
Metastasis	100 (75%)
Elevated pre-treatment LDH	35 (26%)
Bulky tumor	33 (25%)
Large tumor burden	14 (11%)
Pre-existing renal compromise	2 (2%)
NA	21 (16%)
Main Comorbidities	
HTN	14 (11%)
Hepatitis B	10 (8%)
DM	8 (6%)
Dyslipidemia	4 (3%)
COPD	3 (2%)
CKD	3 (2%)
Coronary artery disease	3 (2%)
Other^b^	6 (5%)
NA	94 (71%)

Anti-Cancer Treatment Characteristics

The most common anticancer treatments that induced TLS were chemotherapy (48%; n = 64), targeted therapy (8%; n = 11), and radiotherapy (7%; n = 9). Details of the classes and names are displayed in Table 3.

**Table 3:** Characteristics of the anti-cancer treatments Her2: human epidermal growth factor receptor 2; eGFR: epidermal growth factor receptor; VEGF: vascular endothelial growth factor; CTLA4: cytotoxic T-lymphocyte-associated protein 4; ER: estrogen receptor; PR: progesterone receptor. Due to the use of combination therapies such as chemo-targeted, immune-targeted, and chemo-radiation, some variables may not add up to 100%. ^A^ Others included corticosteroid, eribulin, immunomodulatory therapy (thalidomide), bone-modifying agent (zoledronic acid), and surgery.

Anti-cancer therapy	N (%)
Chemotherapy o Alkylating agents (cisplatin, cyclophosphamide, carboplatin, dacarbazine, oxaliplatin, ifosfamide) o Plant alkaloids (paclitaxel, vincristine, docetaxel, vinblastine, hydroxcamptothecin) o Antimetabolites (fluorouracil, gemcitabine, capecitabine, methotrexate) o Anthracyclines (doxorubicin, epirubicin, adriamycin, mitoxantrone) o Topoisomerase inhibitors (etoposide, irinotecan) o Antibiotics (bleomycin, actinomycin, mitomycin)	64 (48%)
Targeted therapy o Kinase inhibitor (pazopanib, sorafenib, sunitinib, imatinib) o Anti-Her2 (trastuzumab, pertuzumab) o Anti-EGFR (cetuximab) o Anti-VEGF (bevacizumab)	11 (8%)
o Radiotherapy o Radiofrequency ablation	9 (7%)
Immunotherapy o Interleukin-2 o Anti CTLA4 (Ipilimumab) o Autologous lymphocyte therapy	4 (3%)
Hormonal therapy o Anti ER/PR receptors (letrozole) o Anti-androgens (bicalutamide) o Antiestrogen (tamoxifen) o Combined androgen blockade (goserelin acetate)	3 (2%)
Others^A^	7 (5%)

Clinical Manifestations of TLS in Patients with Solid Tumors

TLS occurred spontaneously in 24% (n = 32) of the cases and was treatment-induced in the remaining 76% (n = 100). The number of doses before TLS development was variable, with 17% of the cases showing TLS occurrence after the first dose (n = 23). Time to TLS development was within 3 days of anti-cancer treatment in 37% (n = 49) of the cases, while 52% (n = 68) of the patients received a full dose of anti-cancer treatment. The most commonly reported symptoms were gastrointestinal, genitourinary, and central nervous system symptoms in 33%, 33%, and 26%, respectively. The most reported laboratory abnormalities were hyperuricemia in 95% of the cases (n = 125), followed by elevated creatinine levels in 85% (n = 112) and hyperphosphatemia in 83% (n = 110) of the cases (Table 4).

**Table 4:** Clinical manifestations of TLS in patients with solid tumors TLS: tumor lysis syndrome; GI: gastrointestinal; CNS: central nervous system; GU: genitourinary; NA: not available

TLS manifestation	N (%)
Spontaneous	32 (24%)
Treatment-induced	100 (76%)
Number of doses before TLS	
1	23 (17%)
2-3 or more	5 (4%)
NA	72 (55%)
Time to TLS development	
Spontaneous	32 (24%)
1-2 days	37 (28%)
≥3 days	49 (37%)
NA	13 (10%)
Dose of anti-cancer treatment	
Spontaneous	32 (24%)
Full-dose	68 (52%)
Dose reduction	3 (2%)
NA	29 (22%)
Presenting symptoms	
GI symptoms	44 (33%)
GU symptoms	44 (33%)
CNS symptoms	34 (26%)
Respiratory symptoms	25 (19%)
Constitutional symptoms	15 (11%)
Others	13 (10%)
Cardiovascular Symptoms	11 (8%)
NA	20 (15%)
Presenting laboratory findings	
Elevated uric acid	125 (95%)
Elevated creatinine	112 (85%)
Elevated phosphate	110 (83%)
Elevated LDH	95 (72%)
Elevated potassium	95 (72%)
Low calcium	79 (60%)
Elevated urea	68 (52%)

Management and Clinical Outcomes

Treatment of TLS was mainly based on hydration (58%; n = 77), allopurinol administration (49%; n = 65), and dialysis (30%; n = 40). However, rasburicase use was reported in 24% of patients (n = 32). The majority (77%, n = 101) of the patients were treated in the ward, while 16% (n = 21) were treated in the ICU. More than half (54%, n = 71) of the patients who developed TLS died, and 45% (n = 59) survived (Table 5).

**Table 5:** Management and clinical outcomes in reported cases IVF: intravenous fluid; NA: not available; ICU: intensive care unit; ED: emergency department

Management	N (%)
IVF	77 (58%)
Allopurinol	65 (49%)
Dialysis	40 (30%)
Diuretics	34 (26%)
Rasburicase	32 (24%)
Mechanical ventilation	10 (8%)
Urate oxidase	2 (2%)
NA	16 (12%)
Location	
Ward	101 (77%)
ICU	21 (16%)
ED	10 (8%)
Outcomes	
Dead	71 (54%)
Alive	59 (45%)
NA	2 (2%)

Discussion

Our results showed that males aged 58 years are at higher risk for TLS, which is similar to the findings reported by Mirrakhimov et al. [21]. However, we also observed that hepatocellular carcinoma and lung cancer were the most common cancers, in contrast to the findings reported by Mirrakhimov et al. [21]. This is because our review is more up-to-date and the incidence of TLS in solid tumors is increasing due to advancements in novel anti-cancer treatments [22]. Our review demonstrated that metastatic cancer was a major risk factor for TLS, which is similar to the findings reported by Jallad et al. [23] and Vodopivec et al. [24]. Lastly, chemotherapy was the most common anti-cancer treatment attributed to TLS (48%), as reported by Vodopivec et al. (58%) [24].

To the best of our knowledge, this is the first report to address the manifestations of TLS in solid tumors. TLS occurred spontaneously in 24% of the patients and was induced by the treatment in the remaining 76%. Time to TLS development was ≥3 days following anti-cancer treatment, and 52% of the patients received the full dose of anti-cancer treatment. Additionally, the most commonly reported symptoms were gastrointestinal and genitourinary symptoms in 33% of the patients. The most reported laboratory abnormalities were hyperuricemia (95%), followed by elevated creatinine level (85%), as reported by Vodopivec et al. [24].

In patients with solid tumors who had risk factors for TLS development, large amounts of fluids and allopurinol should be administered before the start of treatment [25]. Once the patient is diagnosed with TLS, treatment should be started using massive amounts of fluids and xanthine oxidase inhibitors such as rasburicase [26]. Our systematic review demonstrated that 58% of patients received intravenous fluids, 49% received allopurinol, and only 24% received rasburicase. These findings illustrate the need for continuous education programs and awareness campaigns to enhance the knowledge of physicians to identify patients at risk and start anti-TLS treatment early and effectively. Moreover, 77% of the patients were treated in the ward, not in the ICU setting. Surprisingly, we found that the mortality rate was 54%, and this is the first report describing the mortality rate associated with TLS in patients with solid tumors. Previous reports evaluating TLS in patients with hematological malignancies described mortality rates ranging from 20% to 30%, with the highest reported rate of 79% in AML patients [27-30].

Our systematic review has several strengths, including the fact that it is the largest and most comprehensive systematic review of case reports describing TLS in patients with solid tumors, manifestations of TLS following anti-cancer treatment, and the most common symptoms. However, our study also has several limitations: an important caveat for interpreting our study findings is the nature of case reports, since authors report unique cases and the findings may not account for unpublished reports of TLS. One inherent weakness of this study is the limited availability of data in case reports. Another important limitation is that the reporting of the drug dosage, number of doses, and schedule was incomplete in several case reports, and we were unable to determine whether the number of doses influenced the incidence of TLS.

We believe that the management of TLS should focus on risk assessment, prophylaxis, and treatment [31]. Aggressive hydration with oral and intravenous fluids should be initiated before the start of anti-cancer treatment, and oral hydration and adequate urine output should be maintained for several days after the completion of the treatment [32]. Urate-lowering agents, such as allopurinol or rasburicase, are recommended for prophylaxis and management of TLS [26]. Febuxostat is also a urate-lowering agent that can provide better control of hyperuricemia in TLS with a good safety profile if allopurinol is contraindicated or not available.

The findings show that TLS is a lethal condition, and early identification with prompt initiation of preventative measures is essential to save patient lives. Although the data indicated modest prognostic benefits, early initiation of anti-TLS measures will improve oncological outcomes. Care of patients with TLS requires an interdisciplinary approach including nephrologists, intensivists, oncologists, and internists in closed observation units, such as intermediate care or ICUs [33,34].

## Conclusions

In this systematic review, we found that older men had a higher tendency to develop TLS. Hepatocellular carcinoma was the most common type of cancer leading to TLS development, followed by lung cancer and melanoma. Metastatic cancer was a contributing risk factor for TLS development. Chemotherapy was the most common class of anti-cancer treatment that induced TLS. Manifestations of TLS developed within ≥3 days following anti-cancer treatment, and half of the patients received the full dose of anti-cancer treatment. Gastrointestinal and genitourinary symptoms were the most commonly reported, and almost all patients showed high uric acid and elevated creatinine levels.

## Appendices

Appendix 1

**Table 6:** Search methodology

Pubmed:	
#	Keywords
1	"tumor lysis syndrome"
2	"spontaneous tumor lysis syndrome"
3	"Acute tumor lysis syndrome"
4	"tumour lysis syndrome"
5	OR/1-4
6	("Solid tumor" OR "Solid cancer" OR "solid carcinoma" OR "solid neoplasm")
7	("breast cancer" OR "breast carcinoma" )
8	("lung cancer" OR "lung carcinoma")
9	("liver cancer" OR "hepatic carcinoma")
10	("ovarian cancer" OR "ovarian carcinoma" OR "ovarian tumor")
11	("colon cancer" OR "colon carcinoma" OR "colon tumor")
12	("gastric cancer" OR "gastric tumor")
13	("Brain cancer" OR "brain tumor")
14	("prostate cancer" OR "prostate tumor")"skin tumor"
15	"skin tumor"
16	sarcoma
17	("bone cancer" OR "bone carcinoma")
18	("pancreatic cancer" OR "pancreatic carcinoma" OR "pancreatic tumor")
19	"cervical cancer"
20	"cervix carcinoma"
21	("endometrial cancer" OR "endometrial tumor" OR "endometrial adenocarcinoma")
22	("esophageal cancer" OR "esophageal tumor")
23	("hepatocellular cancer" OR "hepatocellular carcinoma") ("small cell cancer" OR "small cell carcinoma" OR "small cell tumor")
24	("small cell cancer" OR "small cell carcinoma" OR "small cell tumor")
25	("germ cell cancer" OR "germ cell tumor")
26	osteosarcoma
27	neuroblastoma
28	medulloblastoma
29	("renal cancer" OR "renal carcinoma" OR "renal cell cancer" OR "renal cell carcinoma" OR "renal cell tumor")
30	mesothelioma
31	glioblastoma
32	melanoma
33	OR/ 6-32
34	5 AND 33
35	English
36	Human
37	Adult(+19)
38	OR 35-37
39	34 And 38
Ovid:	
#	Keywords
1	All of resources were selected except books
2	"tumor lysis syndrome"
3	"spontaneous tumor lysis syndrome"
4	"Acute tumor lysis syndrome"
5	"tumour lysis syndrome"
6	OR/2-5
7	("Solid tumor" OR "Solid cancer" OR "solid carcinoma" OR "solid neoplasm")
8	("breast cancer" OR "breast carcinoma" )
9	("lung cancer" OR "lung carcinoma")
10	("liver cancer" OR "hepatic carcinoma")
11	("ovarian cancer" OR "ovarian carcinoma" OR "ovarian tumor")
12	("colon cancer" OR "colon carcinoma" OR "colon tumor")
13	("gastric cancer" OR "gastric tumor")
14	("Brain cancer" OR "brain tumor")
15	("prostate cancer" OR "prostate tumor")
16	"skin tumor"
17	sarcoma
18	("bone cancer" OR "bone carcinoma")
19	("pancreatic cancer" OR "pancreatic carcinoma" OR "pancreatic tumor")
20	"cervical cancer"
21	"cervix carcinoma"
22	("endometrial cancer" OR "endometrial tumor" OR "endometrial adenocarcinoma")
23	("esophageal cancer" OR "esophageal tumor")
24	("hepatocellular cancer" OR "hepatocellular carcinoma")
25	("small cell cancer" OR "small cell carcinoma" OR "small cell tumor")
26	("germ cell cancer" OR "germ cell tumor")
27	Osteosarcoma
28	neuroblastoma
29	medulloblastoma
30	("renal cancer" OR "renal carcinoma" OR "renal cell cancer" OR "renal cell carcinoma" OR "renal cell tumor")
31	mesothelioma
32	glioblastoma
33	Melanoma
34	OR/ 7-33
35	6 AND 34
36	D duplicates from ovid
37	English
38	Human
39	Adult(+19)
40	OR 36-39
41	35 And 40
Cochrane library:	
1	"tumor lysis syndrome"
2	"spontaneous tumor lysis syndrome"
3	"Acute tumor lysis syndrome"
4	"tumour lysis syndrome"
5	OR/1-4
6	("Solid tumor" OR "Solid cancer" OR "solid carcinoma" OR "solid neoplasm")
7	("breast cancer" OR "breast carcinoma")
8	("lung cancer" OR "lung carcinoma")
9	("liver cancer" OR "hepatic carcinoma")
10	("ovarian cancer" OR "ovarian carcinoma" OR "ovarian tumor")
11	("colon cancer" OR "colon carcinoma" OR "colon tumor")
12	("gastric cancer" OR "gastric tumor")
13	("Brain cancer" OR "brain tumor")
14	("prostate cancer" OR "prostate tumor")
15	"skin tumor"
16	sarcoma
17	("bone cancer" OR "bone carcinoma")
18	("pancreatic cancer" OR "pancreatic carcinoma" OR "pancreatic tumor")
19	"cervical cancer"
20	"cervix carcinoma"
21	("endometrial cancer" OR "endometrial tumor" OR "endometrial adenocarcinoma")
22	("esophageal cancer" OR "esophageal tumor")
23	("hepatocellular cancer" OR "hepatocellular carcinoma")
24	("small cell cancer" OR "small cell carcinoma" OR "small cell tumor")
25	("germ cell cancer" OR "germ cell tumor")
26	osteosarcoma
27	neuroblastoma
28	medulloblastoma
29	("renal cancer" OR "renal carcinoma" OR "renal cell cancer" OR "renal cell carcinoma" OR "renal cell tumor")
30	mesothelioma
31	glioblastoma
32	Melanoma
33	OR/ 6-32
34	5 AND 33

Appendix 2

**Table 7:** Quality assessment of included studies

Author	Year	Title	Demographics (age, sex)	Current health status	Medical history	Physical exam	Patient disposition	Drug identification	Dosage	Drug reaction interface	Concomitant therapy	Adverse events	Discussion
Katiman 2012 [2]	Yes	Yes	Yes	Yes	Yes	Yes	Yes	Yes	Yes	Yes	No	Yes	Yes
Kekre 2012 [3]	Yes	Yes	Yes	Yes	Yes	Yes	Yes	No	No	No	No	No	Yes
Mouallem 2013 [4]	Yes	Yes	Yes	Yes	Yes	Yes	Yes	Yes	No	Yes	Yes	Yes	Yes
Durham 2017 [5]	Yes	Yes	Yes	Yes	Yes	Yes	Yes	No	No	No	No	No	Yes
D’Alessandro 2010 [6]	Yes	Yes	Yes	Yes	Yes	Yes	Yes	No	No	No	No	Yes	Yes
Drakos 1994 [7]	Yes	Yes	Yes	Yes	Yes	Yes	Yes	Yes	Yes	Yes	No	Yes	Yes
Tomlinson 1984 [8]	Yes	Yes	Yes	Yes	Yes	Yes	Yes	Yes	Yes	Yes	Yes	Yes	Yes
Marinella 1999 [9]	Yes	Yes	Yes	Yes	Yes	Yes	Yes	Yes	Yes	Yes	No	Yes	Yes
Castro 1999 [10]	Yes	Yes	Yes	Yes	Yes	Yes	Yes	Yes	Yes	Yes	No	Yes	Yes
Han 2008 [11]	Yes	Yes	Yes	Yes	Yes	Yes	Yes	Yes	Yes	Yes	No	Yes	Yes
Lehnar 2005 [12]	Yes	Yes	Yes	Yes	Yes	Yes	Yes	Yes	Yes	Yes	Yes	Yes	Yes
Sklarin 1995 [13]	Yes	Yes	Yes	Yes	Yes	Yes	Yes	No	No	No	No	Yes	Yes
Borne 2009 [14]	Yes	Yes	Yes	No	Yes	Yes	Yes	Yes	Partial	Yes	Yes	Yes	Yes
Hsieh 2009 [15]	Yes	Yes	Yes	Yes	Yes	Yes	Yes	Yes	Yes	Yes	No	Yes	Yes
Kim 2017 [17]	Yes	Yes	Yes	Yes	Yes	Partial	Yes	No	No	No	No	No	Yes
van Kalleveen 2018 [18]	Yes	Yes	Yes	Yes	Yes	Yes	Yes	Yes	Yes	Yes	No	Yes	Yes
Vaidya 2015 [35]	Yes	Yes	Yes	Yes	Yes	Yes	Yes	Yes	Yes	Yes	No	Yes	Yes
Baeksgaard 2003 [25]	Yes	Yes	Yes	Yes	Yes	Yes	Yes	Yes	Yes	Yes	No	Yes	Yes
Farooqi 2015 [36]	Yes	Yes	Yes	Yes	Yes	Yes	Yes	Yes	No	Yes	No	Yes	Yes
Gbaguidi 2016 [37]	Yes	Yes	Yes	Yes	Yes	Partial	Yes	No	NO	NO	NO	NO	Yes
Bilgrami 1993 [38]	Yes	Yes	Yes	Yes	Yes	Yes	Yes	Yes	Yes	Yes	No	Yes	Yes
Camarata 2013 [39]	Yes	Yes	Yes	Yes	Yes	Yes	Yes	Yes	Yes	Yes	Yes	Yes	Yes
Geum 2008 [40]	Yes	Yes	Yes	Yes	Yes	Yes	Yes	Yes	Yes	Yes	No	Yes	Yes
Blanke 2000 [41]	Yes	Yes	Yes	Yes	Yes	Yes	Yes	Yes	Yes	Yes	No	Yes	Yes
Wang 2010 [42]	Yes	Yes	Yes	Yes	Yes	Yes	Yes	Yes	Yes	Yes	No	Yes	Yes
Bhardwaj 2018 [43]	Yes	Yes	Yes	Yes	Yes	Yes	Yes	Yes	Yes	Yes	No	Yes	Yes
Ajzensztejn 2006 [44]	Yes	Yes	Yes	Yes	Yes	Yes	Yes	Yes	Yes	Yes	No	Yes	Yes
Chan 2005 [45]	Yes	Yes	Yes	Yes	No	Yes	Yes	Yes	Partial	Yes	No	Yes	Yes
Baudon 2016 [46]	Yes	Yes	Yes	Yes	Yes	Yes	Yes	Yes	Partial	Yes	No	Yes	Yes
Beriwal 2002 [47]	Yes	Yes	Yes	Yes	Yes	Yes	Yes	Yes	Partial	Yes	No	Yes	Yes
Godoy 2010 [48]	Yes	Yes	Yes	Yes	Yes	Yes	Yes	Yes	No	Yes	No	Yes	Yes
Gongora 2019 [49]	Yes	Yes	Yes	Yes	Yes	Yes	Yes	Yes	Partial	Yes	No	Yes	Yes
Gold 1993 [50]	Yes	Yes	Yes	Yes	Yes	Yes	Yes	Yes	Yes	Yes	No	Yes	Yes
Yoshimura 2008 [51]	Yes	Yes	Yes	No	Yes	Yes	Yes	Yes	No	No	No	yes	No
Boikos 2013 [52]	Yes	Yes	Yes	Yes	No	Yes	Yes	Yes	Partial	Yes	No	Yes	Yes
Dar 2014 [53]	Yes	Yes	Yes	Yes	No	Yes	Yes	Yes	Partial	Yes	No	Yes	Yes
Woo 2001 [54]	Yes	Yes	Yes	Yes	Yes	Yes	Yes	No	No	No	No	No	Yes
Vogelzang 1983 [55]	Yes	Yes	Yes	Yes	Yes	Yes	Yes	Yes	Partial	Yes	No	Yes	Yes
Yahata 2006 [56]	Yes	Yes	Yes	Yes	Yes	Yes	Yes	Yes	Yes	Yes	Yes	Yes	Yes
Chao 2012 [57]	Yes	Yes	Yes	Yes	No	Yes	Yes	No	No	Yes	No	Yes	Yes
Baumann 1983 [58]	Yes	Yes	Yes	Yes	No	Yes	No	Yes	Yes	Yes	No	Yes	Yes
Abbass 2011 [59]	Yes	Yes	Yes	Yes	Yes	Yes	Yes	Yes	Yes	Yes	No	Yes	Yes
Vishwanathan 2019 [60]	No	Yes	Yes	Yes	Yes	Yes	Yes	No	No	No	No	Yes	Yes
Stoves 2001 [61]	Yes	Yes	Yes	Yes	Yes	Yes	Yes	Yes	Yes	Yes	No	Yes	No
Tsai 2012 [62]	Yes	Yes	Yes	Yes	Yes	Yes	Yes	No	No	Yes	No	Yes	Yes
Hiraizumi 2011 [63]	Yes	Yes	Yes	Yes	Yes	Yes	Yes	Yes	Partial	Yes	No	Yes	Yes
Hentrich 2008 [64]	Yes	Yes	Yes	Yes	Yes	Yes	Yes	Yes	Yes	Yes	No	yes	yes
Hussein 1990 [65]	Yes	Yes	Yes	Yes	Yes	Yes	Yes	Yes	Yes	Yes	No	Yes	Yes
Burney 1998 [66]	Yes	Yes	Yes	Yes	Yes	Yes	Yes	Yes	Partial	Partial	No	Yes	Yes
Cihan 2015 [67]	Yes	Yes	Partial	Yes	No	Yes	Yes	Yes	Yes	Yes	No	Yes	Yes
Agarwala 2017 [68]	Yes	Yes	Yes	Yes	Yes	Yes	Yes	No	No	No	No	Yes	Yes
Catania 2017 [69]	Yes	Yes	Yes	Yes	No	Yes	No	No	No	No	No	Yes	Yes
Ignaszewski 2017 [70]	Yes	Yes	Yes	Yes	Yes	Yes	Yes	No	No	No	No	Yes	Yes
Jallad 2011 [23]	Yes	Yes	Yes	Yes	Yes	Yes	Yes	No	No	No	No	Yes	Yes
Stuart 2017 [71]	Yes	Yes	partial	Yes	Yes	Yes	Yes	Yes	No	Yes	No	Yes	Yes
Jiang 2016 [72]	Yes	Yes	Yes	Yes	Yes	Yes	Yes	Yes	Yes	Yes	No	Yes	Yes
Kallab 2001 [73]	Yes	Yes	Yes	Yes	Yes	Yes	Yes	Yes	Yes	Yes	No	Yes	Yes
Kaplan 2012 [74]	Yes	Yes	Yes	Yes	Yes	Yes	Yes	Yes	Yes	Yes	No	Yes	Yes
Sewani 2002 [75]	Yes	Yes	Yes	Yes	Yes	Yes	Yes	Yes	Yes	Yes	No	Yes	Yes
Sakamoto 2007 [76]	Yes	Yes	Yes	Yes	Yes	Yes	Yes	Yes	Yes	Yes	No	Yes	Yes
Taira 2015 [77]	Yes	Yes	Yes	Yes	Yes	Yes	Yes	Yes	No	Yes	No	Yes	Yes
Sorscher 2004 [78]	Yes	Yes	Yes	Yes	Yes	Yes	Yes	Yes	Yes	Yes	No	Yes	Yes
Shiba 2008 [79]	Yes	Yes	Yes	Yes	Yes	Yes	Yes	Yes	Yes	Yes	No	Yes	Yes
Regnault 2016 [80]	Yes	Yes	Yes	Yes	Yes	Yes	Yes	Yes	No	Yes	No	Yes	Yes
Wright 2005 [81]	Yes	Yes	Yes	Yes	Yes	Yes	Yes	No	No	No	No	Yes	partial
Weil 2018 [82]	Yes	Yes	Yes	Yes	Yes	Yes	Yes	No	No	No	No	Yes	Yes
Mazzoni 2016 [83]	Yes	Yes	Yes	Yes	Yes	Yes	Yes	Yes	no	Yes	No	Yes	Yes
Krishnan 2008 [84]	Yes	Yes	Yes	Yes	Yes	Yes	Yes	Yes	Yes	Yes	No	Yes	Yes
Lee 2006 [85]	Yes	Yes	Yes	Yes	Yes	Yes	Yes	Yes	Yes	Yes	No	No	Yes
Saleh 2015 [86]	Yes	Yes	Yes	Yes	Yes	Yes	Yes	No	No	No	No	Yes	Yes
Zigrossi .2001 [87]	No	Yes	No	Yes	No	No	Yes	Yes	No	No	No	Yes	No
Kalemkerian 1997 [88]	Yes	Yes	Yes	Yes	Yes	Yes	Yes	Yes	Yes	Yes	No	Yes	Yes
Habib 2002 [89]	Yes	Yes	Yes	Yes	Yes	Yes	Yes	Yes	Yes	Yes	No	Yes	Yes
Stark 1987 [90]	Yes	Yes	Yes	Yes	Yes	Yes	Yes	Yes	Yes	Yes	No	Yes	Yes
Meeks 2016 [91]	Yes	Yes	Yes	Yes	No	Yes	Yes	Yes	Yes	Yes	No	Yes	Yes
Busam 2004 [92]	Yes	Yes	Yes	Yes	No	Partial	Yes	Yes	No	No	No	Yes	Yes
Mehrzad 2014 [93]	Yes	Yes	Yes	Yes	Yes	Yes	Yes	No	No	No	No	Yes	Yes
Michels 2010 [94]	Yes	Yes	Yes	Yes	Yes	Yes	Yes	Yes	Yes	Yes	No	Yes	Yes
Nakamura 2009 [95]	Yes	Yes	Yes	Yes	No	Yes	Yes	Yes	Yes	Yes	No	Yes	Yes
Gouveia 2018 [96]	Yes	Yes	Yes	Yes	Yes	Yes	Yes	Yes	Partial	No	No	Yes	Yes
Huang 2009 [97]	Yes	Yes	Yes	Yes	Yes	Yes	Yes	Yes	Yes	Yes	No	Yes	Yes
Lin 2007 [98]	Yes	Yes	Yes	Yes	Yes	Yes	Yes	No	No	No	No	Yes	Yes
Nicholaou 2007 [99]	Yes	Yes	Yes	Yes	Yes	Yes	Yes	Yes	No	Yes	No	Yes	Yes
Norberg 2014 [100]	Yes	Yes	Yes	Yes	No	Yes	Yes	No	No	No	No	Yes	Yes
OztopI 2004 [101]	Yes	Yes	Yes	Yes	Yes	Yes	Yes	Yes	Yes	Yes	No	Yes	Yes
Pabon 2018 [102]	Yes	Yes	Partial	Yes	Yes	Yes	Yes	Yes	Yes	Yes	No	Yes	Yes
Pindak 2019 [103]	Yes	Yes	Yes	Yes	Yes	Yes	Yes	Yes	No	Yes	No	Yes	Yes
Rostom 2000 [104]	Yes	Yes	Yes	Yes	Yes	Yes	Yes	Yes	Yes	Yes	No	Yes	Yes
Romo 2019 [105]	Yes	Yes	Yes	No	Yes	Yes	Yes	Yes	Yes	No	No	No	Yes
Okay 2019 [106]	Yes	Yes	Yes	No	Yes	Yes	Yes	Yes	No	partial	No	No	No
Dhakal 2018 [107]	Yes	Yes	Yes	Yes	Yes	Yes	Yes	No	No	No	No	Yes	Yes
Shiozawa 2010 [108]	Yes	Yes	Yes	No	Yes	Yes	No	Yes	Partial	Yes	No	Yes	No
Dirix 1991 [109]	Yes	Yes	Yes	Yes	Yes	Yes	Yes	Yes	Yes	Yes	No	Yes	Yes
Shamseddine. 1993 [110]	Yes	Yes	Yes	Yes	Yes	Yes	Yes	Yes	Yes	Yes	No	Yes	Partial
Song. 2011 [111]	Yes	Yes	Yes	Yes	Yes	Yes	Yes	No	No	No	No	Yes	Yes
Takeuchi 2016 [112]	Yes	Yes	Yes	Yes	Yes	Yes	Yes	No	No	No	No	Yes	Yes
Kim 2014 [113]	Yes	Yes	Yes	Yes	Yes	Yes	Yes	Yes	Partial	Yes	No	Yes	Yes
Chow 2015 [114]	Yes	Yes	Yes	Yes	Yes	Yes	Yes	No	No	No	No	Yes	Yes
Cech 1986 [115]	No	Yes	Yes	Yes	Yes	Yes	Yes	Yes	Yes	Yes	No	Yes	Yes
Feld 2000 [116]	Yes	Yes	Yes	Yes	Yes	Yes	Yes	No	No	No	No	Yes	Yes
Boisseau 1996 [117]	Yes	Yes	Yes	Yes	Yes	Yes	Yes	Yes	Yes	Yes	No	Yes	No
Alaigh 2017 [118]	Yes	Yes	Yes	Yes	Yes	Yes	Yes	Yes	Yes	Yes	No	Yes	Yes
Pinder 2007 [119]	Yes	Yes	Yes	Yes	Yes	Yes	Yes	Yes	Yes	Yes	No	Yes	Yes
Kurt 2005 [120]	Yes	Yes	Yes	No	No	Yes	Yes	Yes	Yes	Yes	Yes	Yes	Yes
Kawai 2006 [121]	Yes	Yes	Yes	Yes	No	Yes	Yes	Yes	No	Yes	No	Yes	Yes
Vaisban 2003 [122]	Yes	Yes	Yes	Yes	Yes	Yes	Yes	No	No	No	No	Yes	Yes
Okamoto 2015 [123]	Yes	Yes	Yes	Yes	Yes	Yes	Yes	No	No	No	No	Yes	Yes
Boyd 2017 [124]	Yes	Yes	Yes	Yes	Yes	Yes	Yes	No	No	No	No	Yes	Yes
Vodopivec 2012 [24]	Yes	Yes	Yes	Yes	Yes	Yes	Yes	Yes	No	Yes	No	Yes	Yes
Tseng 2016 [125]	Yes	Yes	Yes	Yes	Yes	Yes	Yes	Yes	Yes	Yes	No	Yes	Yes
Berringer 2017 [126]	Yes	Yes	Yes	Yes	Yes	Yes	Yes	No	No	No	No	Yes	Yes
Sommerhalder 2017 [127]	Yes	Yes	Yes	Yes	Yes	Yes	Yes	No	No	No	No	Yes	Yes
Shenoy 2009 [128]	Yes	Yes	Yes	Yes	Yes	Yes	Yes	No	No	No	No	Yes	Yes
Lee 2013 [129]	Yes	Yes	Yes	Yes	Yes	Yes	Yes	Yes	Yes	Yes	No	Yes	Yes
Tanvetyanon 2004 [130]	Yes	Yes	Yes	Yes	Yes	Yes	Yes	Yes	Yes	Yes	No	No	No
Ustundag 1997 [131]	Yes	Yes	Yes	Yes	Yes	Yes	Yes	Yes	Partial	Yes	No	Yes	Yes
Abbouda 2009 [132]	Yes	Yes	Yes	Yes	Yes	Yes	Yes	Yes	Yes	Yes	No	Yes	Yes
Barton 1989 [133]	Yes	Yes	Yes	Yes	Yes	Yes	Yes	Yes	Yes	Yes	No	Yes	Yes
Mott 2005 [134]	Yes	Yes	Yes	Yes	Yes	Yes	Yes	Yes	Partial	Yes	No	Yes	Yes
Qian 2009 [135]	Yes	Yes	Yes	Yes	Yes	Yes	Yes	Yes	Yes	Yes	No	Yes	Yes
Yuan 2017 [136]	Yes	Yes	Yes	Yes	No	Yes	Yes	Yes	Yes	Yes	No	Yes	Yes
Lin 2007 [137]	Yes	Yes	Yes	Yes	Yes	Yes	Yes	Yes	Partial	Yes	No	Yes	Yes
Liang 2012 [138]	Yes	Yes	Yes	Yes	Yes	Yes	No	Yes	Partial	Yes	No	Yes	Yes
Sharma 2006 [139]	Yes	Yes	Yes	Yes	Yes	Yes	Yes	Yes	Yes	Yes	No	Yes	Yes

Appendix 3

**Table 8:** Characteristics of included studies HCC: hepatocellular carcinoma; TACE: trans arterial chemoembolisation; HTN: hypertension; DM: diabetes mellitus; CKD: chronic kidney disease; LDH: lactate dehydrogenase; RCC: renal cell carcinoma; NSCLC: non-small cell lung cancer; COPD: chronic obstructive pulmonary disease; SCLC: small cell lung cancer; PVE: portal vein embolization; ESOS: extraskeletal osteosarcoma; SOB: shortness of breath; BAC: bronchioloalveolar carcinoma; TLS: tumor lysis syndrome TURP: transurethral resection of the prostate; IMRT: intensity-modulated radiation therapy; GIST: gastrointestinal stromal tumor

Case (Author, year, (reference no.)	Age (years)	Gender (M/F)	Primary cancer	Anti-cancer treatment: Full dose or reduced (class and name)	Number of doses: days preceding presentation	Any comorbidities	Presenting symptoms	Risk factors
Katiman, 2012 [2]	55	M	HCC	chemotherapy: TACE (doxorubicin)	1 cycle:1 dose: 1 day after initiation	HTN, hepatitis B	right hypochondrial pain, nausea, haematuria.	bulky tumor
Kekre, 2012 [3]	76	M	HCC	spontaneous	none	hemochromatosis, arthritis, DM, CKD, HTN, dyslipidemia, erectile dysfunction.	nausea, vomiting, diarrhea, epigastric pain, and decreased appetite.	bulky tumor, pre-existing renal compromise.
Mouallem, 2013 (Case 1) [4]	68	M	melanoma	chemotherapy: dacarbazine	3 courses: 3 days after the last course	N/A	nausea, vomiting, weakness, confusion and oliguria	bulky and metastatic tumor
Mouallem, 2013 (Case 2) [4]	69	M	melanoma	spontaneous	N/A	N/A	rectal bleeding	bulky and metastatic tumor
Durham, 2018 [5]	59	M	melanoma	spontaneous	N/A	N/A	abdominal pain, nausea	metastatic tumor
D’Alessandro, 2010 [6]	22	M	germ cell tumor	spontaneous	N/A	DM	abdominal fullness, epigastric pain, weight loss and lethargic	metastatic tumor
Drakos, 1994 [7]	32	F	breast carcinoma	Chemotherapy: mitoxantrone 14 mg/m2: 22 mg, full dose	1 cycle: 2 doses: 4 days after initiation.	N/A	nausea, vomiting, abdominal pain, confusion	rapidly growing tumors with spreading to other organ, and pretreatment high LDH
Tomlinson, 1984 [8]	34	F	medulloblastoma	Radiotherapy: cobalt-60 100 radiation per day, full dose	fourth day after total of 300 radiation	N/A	oliguria	metastatic cancer, pretreatment high LDH
Marinella, 1999 [9]	52	M	SCLC	Chemotherapy: etoposide (100mg/m2) and cisplatin 30 mg/m2), full dose	1 cycle: 1 days after initiation	DM and HTN	lethargic, hematochezia	metastatic tumor
Castro, 1999 [10]	61	M	melanoma	Biochemotherapy: interleukin-2 MIU/M2/Day IV, interferon-𝝰 5MU/M2/day SQ, dacarbazine 800mg/m2/day, vinblastine 1.6mg/m2/day IV, cisplatin 20mg/m2/day IV, full dose	1 cycle: 4 days after initiation	N/A	oliguria	metastatic and bulky tumor
Han, 2008 [11]	38	M	gastric cancer	Chemotherapy: capecitabine 1,250 mg/m2 orally twice daily on day 1 through 14, plus cisplatine 60 mg/m2 IV on day 1, full dose	1 cycle: 3 days after initiation	N/A	dyspnea and oliguria	bulky and metastatic tumor, pretreatment high LDH and the tumor is highly sensitive to chemotherapy
Lehnar, 2005 [12]	64	M	HCC	radiofrequency ablation	2 portions of ablation: 2 days after initiation	hepatitis C, DM	hypoxia dyspnea, oliguric, arrhythmia	bulky
Sklarin. 1995 [13]	62	F	breast cancer	spontaneous	N/A	NA	dyspnea	metastatic cancer, high baseline LDH
Borne, 2009 [14]	42	M	melanoma a	corticosteroid high dose	48 hours after initiation	N/A	N/A	metastatic cancer, bulky tumor, pretreatment high LDH
Hsieh (case 1), 2009 [15]	76	F	HCC	chemotherapy: TACE with 20 mg adriamycin, full dose	1 cycle: 1 dose : 3 days after TACE started	N/A	acute renal insufficiency	N/A
Hsieh (case 2), 2009 [15]	56	M	HCC	chemotherapy: TACE with 10 mg of lipiodol + 20 mg adriamycin, full dose	1 cycle: 1 dose: same night of TACE initiation.	hepatitis B	oliguria	N/A
Kim, 2017 [17]	35	F	cervical cancer	spontaneous	N/A	N/A	general weakness	N/A
van Kalleveen, 2018 [18]	58	M	RCC	targeted therapy: pazopanib 800mg, full dose	1 cycle: 6 days after administration	N/A	nausea, vomiting and diarrhea	metastatic cancer
Vaidya, 2015 [35]	52	F	breast cancer	chemotherapy: paclitaxel 80mg/m2, full dose	1 cycle: 1 dose: 1 week after administration	N/A	confusion and sluggishness	metastatic cancer
Baeksgaard, 2003 [25]	23	M	medulloblastoma	chemotherapy (cisplatin 20mg/m2, etoposide 50mg/m2) for five days every 3 weeks full dose	1 cycle 2 dose 2 days after initiation	N/A	fatigue, difficulty in breathing, and low urine output	pretreatment high LDH, and metastatic cancer
Farooqi 2015 [36]	52	M	colorectal cancer (cecum)	targeted therapy (regorafenib)	1 week after initiation	HTN, asthma, and recent stroke	nausea, and vomiting.	metastatic tumor
Gbaguidi, 2016 [37]	88	F	RCC	spontaneous	N/A	HTN, heart failure, and CKD	vomiting	bulky and metastatic tumor, acute medical condition (infection).
Bilgrami , 1993 [38]	47	F	Advanced ovarian cancer	combination chemotherapy: carboplatin 400mg/m2 and cyclophosphamide 500mg/m2, full dose	1 cycle: 1 dose: 4 days after initiation	N/A	N/A	bulky and rapidly growing tumors
Camarata, 2013 [39]	63	F	serous ovarian cancer	combination chemotherapy: carboplatin and paclitaxel 75mg/m2, full dose	1 cycle: 1 dose: 2 days after initiation	high output heart failure	N/A	bulky and metastatic tumor
Geum, 2008 [40]	52	M	NSCLC	palliative radiotherapy: total dosage of 30 Gy divided by 10 fractions, full dose	second fractions (total of 6Gy)	N/A	oliguria, dyspnea	N/A
Blanke, 2000 [41]	52	M	Choriocarcinoma	Chemotherapy: etoposide 100mg/m2 and cisplatin 20mg/m2, full dose	1 cycle: 2 days after initiation	HTN, osteoarthritis, hypercholesterolemia mia	oliguria	metastatic cancer, pretreatment high LDH
Wang, 2010 [42]	54	F	HCC	chemotherapy: TACE with doxorubicin 60 mg and lipiodol 20ml, full dose	1 cycle: 1 dose: 5 days after initiation.	N/A	decreased urine output	N/A
Bhardwaj, 2018 [43]	67	M	prostatic cancer	Chemotherapy: docetaxel 75 mg/m2, full dose	1 cycle: 1 dose 3 days after initiation	N/A	N/A	metastatic tumor
Ajzensztejn, 2006 [44]	65	M	NSCLC	Chemotherapy: docetaxel 75 mg/m2, full dose	1 cycle: 1 dose 3 days after initiation	COPD	drowsiness, breathless, hypotension,acute renal failure	metastatic cancer, and large tumor burden
Chan, 2005 [45]	62	F	ovarian cancer	Chemotherapy: topotecan	2 cycle 2 weeks after initiation	N/A	abdominal pain, nausea, and anorexia	metastatic cancer, large tumor burden, rapid growth tumor, pretreatment high LDH, and bulky
Baudon, 2016 [46]	58	F	breast cancer	target therapy: trastuzumab, pertuzumab	1 cycle: 2 days after her first course	TB	hypovolemic shock	pretreatment high LDH, metastatic cancer, and bulky disease
Beriwal,2002 [47]	68	F	SCLC	chemotherapy: topotecan	1 cycle:1 dose 1 day after initiation	N/A	low urinary output 200ml	pretreatment high LDH and metastatic cancer
Godoy, 2010 [48]	60	F	endometrial cancer	chemotherapy: carboplatin, paclitaxel	1 cycle: 4 days after initiation	N/A	shortness of breath, weakness, and fatigue	metastatic cancer
Gongora, 2019 [49]	46	M	prostatic cancer	chemotherapy: carboplatin, etoposide	5 days after initiation	N/A	N/A	metastatic cancer, pretreatment high LDH
Gold 1993 [50]	66	M	gastric leiomyosarcoma	chemotherapy: cyclophosphamide 2 g/m2; immunotherapy: autolymphocyte therapy, full dose	1 cycle: 1 dose: 16 hours after initiation of the adaptive chemo-immunotherapy	HTN	nausea, fever, and abdominal pain	Large tumor burden and metastatic cancer
Yoshimura, 2008 [51]	59	M	gastric cancer	Chemotherapy: irinotecan and cisplatin reduced dose	After second cycle of chemotherapy	N/A	mild edema of the legs	N/A
Boikos, 2013 [52]	70	F	SCLC	Chemotherapy: cisplatin, etoposide	1 cycle: 8 days after initiation	N/A	N/A	N/A
Dar, 2014 [53]	65	M	melanoma	palliative radiotherapy	5 radiation sessions 7 days after the last session	N/A	general illness, renal insufficiency	metastatic cancer
Woo, 2001 [54]	36	M	gastric cancer	spontaneous	N/A	N/A	abdominal fullness and pain	metastatic cancer
Vogelzang, 1983 [55]	57	F	SCLC	Chemotherapy: doxorubicin50% dose reduction, cisplatin, etoposide, and vincristine sulfate	1 cycle: 1 dose: 36 hours after initiation	N/A	respiratory distress	metastatic cancer
Yahata, 2006 [56]	53	F	ovarian cancer	chemotherapy: paclitaxel 100mg, full dose	5 days after administration	N/A	oliguria	N/A
Chao, 2012 [57]	51	M	HCC	chemotherapy: TACE; type of drugs use not mentioned	N/A	hepatitis B	abdominal pain, oliguria, and fever	N/A
Baumann, 1983 [58]	78	M	SCLC	Chemotherapy: doxorubicin 30mg/sq, cyclophosphamide 900mg/sq, vincristine 2.0mg, full dose	7 days after initiation	N/A	oliguria	metastatic cancer
Abbass, 2011 [59]	62	M	HCC	chemotherapy: sorafenib 800 mg/day, full dose	7 days after initiation	hepatitis B	somnolent, and confused	pretreatment high LDH
Vishwanathan, 2019 [60]	60	F	uterine cancer	spontaneous	N/A	N/A	fatigue, weakness, abdominal girth and pain, anorexia, vaginal spotting, and hematuria	N/A
Stoves, 2001 [61]	43	M	melanoma	chemotherapy and immunotherapy: cisplatin 30mg/m2 and dacarbazine 250mg/m2on days 1-3 and interferon alpha 10MU/m2 on days 1-5 of treatment, full dose	1 cycle: 2 days after initiation	N/A	oliguria, ascites, and edema	metastatic cancer
Tsai, 2012 [62]	51	M	HCC	chemotherapy: PVE and TACE; name of drugs and doses not mentioned	1 cycle: 2 days after initiation	hepatitis B	N/A	large tumor burden
Hiraizumi, 2011 [63]	36	F	uterine leiomyosarcoma	Chemotherapy: vincristine, actinomycin-D, and cyclophosphamide	2 cycle: 7 day after the second cycle of chemotherapy	N/A	confused and decreased urine output	large, bulky and metastatic cancer, pretreatment high LDH
Hentrich, 2008 [64]	62	M	colon cancer	chemotherapy and target therapy: bevacizumab 5 mg/kg IV , irinotecan 50 mg/m2, 5-FU 1400 mg/m2 as 24-hour continuous infusion, and folinic acid 400 mg/m2), full dose	1 cycle: two days after treatment started	N/A	N/A	metastatic cancer and pretreatment high LDH
Hussein, 1990 [65]	57	M	SCLC	chemotherapy: cyclophosphamide 750 mg/m2, doxorubicin 45 mg/m2, and vincristine 2 mg (all intravenously), full dose	1 cycle: 4 days after chemotherapy started	N/A	N/A	metastatic cancer, pretreatment high LDH
Burney, 1998 (case 1) [66]	44	M	HCC	Chemotherapy: TACE (cisplatin 60 mg/m2), full dose	1 cycle:1 dose 8 hours after infusion	N/A	oliguria	N/A
Burney, 1998 (case 2) [66]	46	M	HCC	chemotherapy: TACE, drugs not mentioned	N/A	N/A	N/A	N/A
Cihan, 2015 [67]	61	M	unknown primary tumor	chemotherapy: cetuximab 400mg/m2, irinotecan 125mg/m2, full dose	1 cycle: 1 dose 16 hours after infusion	N/A	N/A	metastatic cancer
Agarwala, 2017 [68]	26	F	HCC	spontaneous	N/A	hepatitis B	abdominal pain, jaundice, and abdominal distention, oliguria	metastatic cancer
Catania, 2017 [69]	65	F	ESOS	spontaneous	N/A	N/A	abdominal pain	metastatic cancer
Ignaszewski, 2017 [70]	69	M	prostate adenocarcinoma	spontaneous	N/A	HTN, hyperlipidemia	nausea, vomiting,weakness, dizziness, and abdominal pain	metastatic cancer
Jallad, 2011 [23]	75	F	SCLC	spontaneous	N/A	COPD, coronary artery disease	SOB, poor appetite, fatigue, increase abdominal girth	high tumor burden and metastatic cancer
Stuart, 2017 [71]	Mid dle Age	M	BAC	palliative radiotherapy	TLS appeared 3 days following radiotherapy	N/A	seizure and global weakness	Metastatic cancer
Jiang, 2016 [72]	52	M	HCC	chemotherapy: TACE (iodised oil 20 ml with 5-fluorouracil 500 ml, epirubicin 30 mg)	1 cycle:1 dose: 1 day after TACE	liver cirrhosis and chronic hepatitis B virus	abdominal pain, fever, and anuric	N/A
Kallab, 2001 [73]	61	M	SCLC	Chemotherapy: cisplatin 80 mg/m2 on day 1 and etoposide 120 mg/m2 on day 1-3, full dose	1 cycle: 1 dose of cisplatin and 3 doses of etoposide 4 days after initiation of chemotherapy	N/A	severe lethargy, oliguria, tachycardia, and hypotension	large tumor burden, metastatic cancer, and pretreatment high LDH
Kaplan, 2012 [74]	60	M	prostate cancer	palliative radiotherapy: total of 30 Gy radiotherapy in 10 fractions, full dose	Day 3 of radiotherapy	N/A	oliguria and dyspnea	metastatic cancer, pretreatment high LDH
Sewani, 2002 [75]	55	M	mixed SCLC and NSCLC	Chemotherapy: carboplatin 830 mg, paclitaxel 440 mg, full dose	1 cycle: 1 dose: 1 day following administration	N/A	Abdominal pain and fever	metastatic cancer
Sakamoto, 2007 [76]	55	M	HCC	chemotherapy: TOCE (15 mL of iodized oil, 50 mg of epirubicin hydrochloride, and embolization with two sheets of gelatin sponge	2 cycles: 1 dose: 1 day following TOCE	hepatitis B	fever, decrease urine output , severe diarrhea, anuria cough, hemoptysis, and dyspnea,	bulky tumor with high LDH level before the TOCE
Taira, 2015 [77]	69	F	breast cancer	targeted therapy: trastuzumab	1 cycle: 6 days following administration	N/A	cardiac arrhythmia	-metastatic cancer
Sorscher, 2004 [78]	80	M	Prostate cancer	chemotherapy: docetaxel at 35 mg/m2) full dose	1 cycle: 1 dose: 1 day after administration	N/A	N/A	-Metastatic cancer, high baseline LDH
Shiba, 2008 [79]	77	M	HCC	chemotherapy: TACE (hydrochloric acid epirubicin 70 mg, 20 mL of iodized oil esters, and 160 mg of porous gelatine grains, full dose	1 cycle: 1 dose: 3 days after administration	N/A	fatigue, fever and oliguria	large tumor burden
Regnault, 2016 [80]	73	M	nodular melanoma	Immunotherapy: Ipilimumab	1 cycle: TLS appears 6 days after initiation	N/A	cardiac arrhythmia	Metastatic cancer, high pretreatment LDH
Wright, 2005 [81]	60	M	prostate cancer	chemotherapy: paclitaxel 100 mg/m2, full dose	1 cycle: 1 dose: 1 day after initiation	N/A	anuria	metastatic cancer
Weil, 2018 [82]	64	F	small cell carcinoma of the cervix	spontaneous	N/A	DM, HTN, dyslipidemia.	weakness, fatigue and abdominal pain	high pretreatment LDH and metastatic cancer
Mazzoni, 2016 [83]	62	M	prostate cancer	palliative radiotherapy: external beam radiation therapy, TURP, and hormonal therapy (bicalutamide)	N/A	N/A	fatigue, weakness, confusion and anuric	metastatic cancer
Krishnan, 2008 [84]	64	M	colon cancer	targeted therapy : cetuximab 400mg/m2, full dose	1 cycle: 1 dose: 18 hours after initiation	N/A	N/A	metastatic cancer
Lee, 2006 [85]	62	M	HCC	immuno-target therapy: thalidomide 300mg per day, full dose	1 cycle: 5 days after initiation	N/A	SOB	N/A
Saleh, 2015 [86]	56	F	pancreatic cancer	spontaneous	N/A	N/A	generalised weakness	metastatic cancer
Zigrossi, 2001 [87]	N/A	F	breast cancer	hormonal therapy: letrozole	N/A	N/A	shock, bilateral pleural effusion, cardiac tamponade, and oliguria	N/A
Kalemkerian, 1997 [88]	74	F	SCLC	Chemotherapy: cisplatin 80 mg/m2 on day 1 and etoposide 100 mg/m2 on days 1-3, full dose.	1 cycle: 1 dose cisplatin and 3 doses etoposide 3 days after chemotherapy initiation	DM	lethargic and oliguric	metastatic tumor and pretreatment high LDH
Habib, 2002 [89]	56	F	melanoma	Steroid: hydrocortisone 100 mg, full dose	2 doses of hydrocortisone: 7 hours after steroid started	N/A	weakness and malaise	metastatic cancer and pretreatment high LDH
Stark, 1987 [90]	53	F	breast adenocarcinoma	Chemotherapy: fluorouracil 400 mg/m2, doxorubicin 40mg/m2,cyclophos phamide 400mg/m2, full dose	N/A	N/A	SOB	metastatic cancer, rapidly growing tumors, pretreatment high LDH, and high tumor burden
Meeks, 2016 [91]	46	M	unknown primary cancer	Steroid: dexamethasone 4mg per 6 hours, full dose	2 days after initiation	anemia	lower back pain	metastatic cancer and bulky tumor
Krishnan, 2008 [84]	64	M	colon cancer	targeted therapy : cetuximab 400mg/m2, full dose.	1 cycle : 1 dose : 18 hours after initiation	N/A	N/A	metastatic cancer
Lee, 2006 [85]	62	M	HCC	immuno-target therapy: thalidomide 300mg per day, full dose	1 cycle: 5 days after initiation	N/A	SOB	N/A
Saleh, 2015 [86]	56	F	pancreatic cancer	spontaneous	N/A	N/A	generalised weakness	metastatic cancer
Zigrossi, 2001 [87]	N/A	F	breast cancer	hormonal therapy: letrozole	N/A	N/A	shock, bilateral pleural effusion, cardiac tamponade, and oliguria	N/A
Kalemkerian, 1997 [88]	74	F	SCLC	chemotherapy: cisplatin 80 mg/m2 on day1 and etoposide 100 mg/m2 on days 1 to 3, full dose	1 cycle: 1 dose cisplatin and 3 doses etoposide 3 days after chemotherapy initiation	DM	lethargic and oliguric	metastatic tumor and pretreatment high LDH
Habib, 2002 [89]	56	F	melanoma	Steroid: hydrocortisone 100 mg, full dose	2 doses of hydrocortisone: 7 hours after steroid started	N/A	weakness and malaise	metastatic cancer and pretreatment high LDH
Stark, 1987 [90]	53	F	breast adenocarcinoma	Chemotherapy: fluorouracil 400 mg/m2, doxorubicin 40mg/m2, cyclophos phamide 400mg/m2, full dose	N/A	N/A	SOB	metastatic cancer, rapidly growing tumors, pretreatment high LDH, and high tumor burden
Meeks, 2016 [91]	46	M	unknown primary cancer	Steroid: dexamethasone 4 mg per 6 hours, full dose	2 days after initiation	anemia	lower back pain	metastatic cancer and bulky tumor
Busam, 2004 [92]	36	F	melanoma	bio-chemo therapy: cisplatin, vinblastine, dacarbazine, interferon-𝝰, interleukin-2	N/A	N/A	N/A	metastatic cancer
Mehrzad, 2014 [93]	70	M	HCC	spontaneous	N/A	withdrawal seizure and HTN	oliguria	bulky tumor and metastatic tumor
Michels, 2010 [94]	48	M	RCC	Targeted therapy: sunitinib 50 mg daily for 4 weeks	Day 18 after initiation of treatment	N/A	fever, headache, vomiting	bulky tumor and metastatic tumor
Nakamura, 2009 [95]	58	M	melanoma	Chemotherapy: cisplatin 70Mg/m2, full dose	N/A	N/A	weakness and malaise	metastatic tumor and bulky
Gouveia, 2018 [96]	51	F	colorectal cancer	palliative chemotherapy: oxaliplatin 85 mg/m2, 5-fluoroucil 400 mg/m2 bolus, 2400 mg/m2 continuous infusion, full dose	after completing three cycles	HTN, obesity	asthenia, fidgetiness, fine tremor.	metastatic tumor. pretreatment high LDH
Huang, 2009 [97]	55	M	HCC	target therapy: sorafenib 400 mg twice every day, full dose.	30 days after sorafenib started	hepatitis B	jaundice, oliguria, weakness	N/A
Lin, 2007 [98]	72	M	prostate carcinoma	spontaneous	N/A	N/A	anorexia, fatigue, and severe pedal edema	metastatic tumor
Nicholaou, 2007 [99]	67	F	RCC	targeted therapy: sunitinib	between days 3-9 of treatment	N/A	watery stools, nausea, vomiting, and fatigue	metastatic tumor
Norberg, 2014 [100]	56	M	RCC	spontaneous	N/A	HTN	severe back pain, night sweets, weight loss and low-grade fevers	metastatic tumor
OztopI, 2004 [101]	66	M	colon cancer	Chemotherapy: irinotecan180 mg/m2 5-fluorouracil 400 mg/m2 bolus 600 mg/m2 continuous infusion leucovorin 200mg/m2, full dose	1 cycle: 72 hours after initiation	N/A	oliguria	metastatic tumor
Pabon, 2018 [102]	mid dle age d	F	uterine leiomyosarcoma	Chemotherapy: ribulin mesylate 1.4mg/m2, full dose	cycle 1 : day 8 after initiation	N/A	fatigue, dyspnoea, and poor appetite	metastatic tumor and bulky tumor
Pindak, 2019 [103]	19	M	testicular germ cell tumor	surgery: radical resection of the tumor	during the surgery	N/A	cardiac arrhythmia	bulky tumor and metastatic tumor
Rostom, 2000 [104]	73	M	breast cancer	radiotherapy: upper hemi-body radiation (UHBI) total breast dose 9.65 Gy, full dose	48 hours after initiation	DM	drowsy, confused	metastatic tumor
Romo, 2019 [105]	28	M	oligodendroglioma	Radiotherapy: IMRT with a cumulative dose of 5940 cGy over 33 fractions	N/A	N/A	N/A	metastatic tumor and rapidly growing tumor
Okay, 2019 [106]	61	M	HCC	chemotherapy: TACE (ethanol and lipiodol)	2 weeks after initiation	chronic myeloid leukemia	N/A	N/A
Dhakal, 2018 [107]	70	M	small cell neuroendocrine carcinoma	spontaneous	N/A	coronary artery disease	fatigue, leg swelling, heartburn, nausea, abdominal pain, decreased urinary output	metastatic
Shiozawa, 2010 [108]	79	F	HCC	targeted therapy: sorafenib	1 cycle: 10 days after initiation	hepatitis C and liver cirrhosis	N/A	N/A
Dirix, 1991 [109]	65	F	Merkle cell carcinoma	chemotherapy: doxorubicin 50mg/m2 IV bolus, and 5 g/m2 continuous infusion over 24 hours of ifosfamide, full dose	1 cycle: 4 days after chemotherapy initiation	N/A	anuria.	bulky and metastatic tumor, highly sensitive to chemotherapy, and pretreatment high LDH
Shamseddine, 1993 [110]	66	F	valvular cancer	chemotherapy: cisplatin, 50 mg as continuous infusion over 4 hours daily for 3 days, 5 FU, 1500 mg as continuous infusion over 24 hours for 5 days, full dose.	1 cycle	N/A	tachypnea and sweating	bulky and metastatic tumor
Song, 2011 [111]	46	M	melanoma	spontaneous	N/A	N/A	abdominal pain, nausea, vomiting, and dyspnea	metastatic tumor, large tumor burden, high tumor proliferation rate, elevated serum LDH
Takeuchi, 2016 [112]	62	M	melanoma	spontaneous	N/A	DM	oliguria and back pain	metastatic tumor and elevated serum LDH
Kim, 2014 [113]	59	M	colon cancer	chemotherapy: 5-FU, leucovorin, and oxaliplatin	2nd cycle : 3 days after chemotherapy, and 3rd cycle: 3 days after chemotherapy.	N/A	N/A	metastatic tumor
Chow, 2015 [114]	47	M	testicular cancer	spontaneous	N/A	N/A	breathlessness, bilateral limb swelling, and tachypnea.	N/A
Cech, 1986 [115]	94	F	breast cancer	hormonal therapy: tamoxifen 10mg by mouth twice a day, full dose	one week after initiation	N/A	bone pain	metastatic cancer
Feld, 2000 [116]	72	M	lung adenocarcinoma	spontaneous	N/A	N/A	increasing abdominal girth, jaundice, fever, weight lose, and night sweats	high baseline LDH and metastatic
Boisseau, 1996 [117]	42	F	colon cancer	Chemotherapy: irinotecan 300mg/m2, reduced dose	8 days after initiation	N/A	general deterioration	metastatic, bulky, and rapid growth tumor
Alaigh, 2017 [118]	58	F	leiomyosarcoma	spontaneous	N/A	N/A	abdominal distention, constipation, nausea, fatigue, and SOB	metastatic
Pinder, 2007 [119]	81	M	gastrointestinal stromal tumor (GIST)	target therapy: imatinib 400mg once daily, full dose	2 days after initiation	N/A	SOB, oedema, and poor urine output	metastatic and bulky tumor
Kurt, 2005 [120]	52	M	lung adenocarcinoma	bone modifying therapy: zoledronic acid 4mg infused within 15 minutes	4 days after initiation	N/A	N/A	metastatic, bulky and large tumor border
Kawai, 2006 [121]	26	M	testicular cancer	chemotherapy: bleomycin, etoposide and cisplatin (BEP)	1 day after initiation	N/A	Day 2 abdominal pain, Day 4 Massive melena	metastatic and pretreatment high LDH
Vaisban, 2003 (case 1) [122]	82	F	colon cancer	spontaneous	N/A	N/A	weakness, oliguria, and confusion	metastatic tumor
Vaisban, 2003 (case 2) [122]	80	M	pheochromocytoma	spontaneous	N/A	N/A	abdominal pain, fever, and vomiting	N/A
Vaisban, 2003 (case 3) [122]	72	M	HCC	spontaneous	N/A	N/A	abdominal pain, dyspnea, and weakness	N/A
Okamoto, 2015 [123]	62	F	ovarian cancer	spontaneous	N/A	N/A	lower abdominal pain, back pain, and anuria	bulky
Boyd, 2017 [124]	56	M	prostate cancer	spontaneous	N/A	N/A	abdominal pain	metastatic and bulky tumor, high LDH level, and pre-existing renal disease.
Vodopivec, 2012 [24]	57	M	gastric adenocarcinoma	chemotherapy: oxaliplatin, docetaxel, floxuridine, and leucovorin	7 days after initiation of first cycle	N/A	Nausea, vomiting, oliguria, and generalized weakness	metastatic
Tseng, 2016 [125]	65	M	colon cancer	chemotherapy: oxaliplatin 160 mg (85 mg/m2), 5-FU 2800 mg (1500 mg/m2) for 1 day, full dose	1st cycle: 4 days after chemotherapy started	N/A	chest tightness, altered level of consciousness, and ventricular tachycardia.	metastatic tumor
Berringer, 2017 [126]	48	M	colon cancer	spontaneous	N/A	N/A	abdominal pain,jaundice, weakness, and anorexia.	metastatic tumor and large tumor burden
Sommerhalder, 2017 [127]	49	F	colon cancer	spontaneous	N/A	HTN and anemia	edema of bilateral extremities associated with worsening dyspnea	metastatic tumor
Shenoy, 2009 [128]	74	M	SCLC	spontaneous	N/A	COPD, coronary artery disease, and HTN	anuria, lethargy, and weakness	bulky
Lee, 2013 [129]	40	F	thymoma	chemotherapy: IV paclitaxel 175mg/m2, IV ifosfamide 2500mg/m2, full dose	second day of chemotherapy	N/A	tachypnea, tachycardia, and oliguria	bulky, pretreatment high LDH, and metastatic tumor
Tanvetyanon, 2004 [130]	77	M	prostate cancer	hormonal therapy: goserelin acetate 10.8mg, full dose	6 days after initiation hormonal therapy	N/A	Lethargic and flapping tremor	bulky, pretreatment high LDH, and metastatic tumor
Ustundag, 1997 [131]	56	F	breast cancer	chemotherapy: paclitaxel IV infusion for 24 hours, full dose	one day after initiation	N/A	orthopnea, oliguria, and anuria	Metastatic cancer, high pretreatment LDH
Abbouda, 2009 [132]	53	M	maxillary sinus cancer	adjuvant chemoradiation: 66 Gy to the tumor bed and 50 Gy to the upper neck area, full dose	4 days after initiation	N/A	decreased level of consciousness and abdominal pain.	metastatic cancer
Barton. 1989 (case 1) [133]	57	F	breast cancer	chemotherapy: cyclophosphamid e 500mg/m2, methotrexate 30mg/m2 and 5-fluorouracil 500mg/m2, full dose	1 day after initiation	N/A	dyspnea	bulky, metastatic tumor, pretreatment high LDH, rapid tumor growth, and large tumor border
Barton, 1989 (case 2) [133]	58	M	seminoma	chemotherapy: vinblastine 0.2 mg/kg/d and IV bleomycin 30 units daily, full dose	2 days after initiation	N/A	N/A	bulky, metastatic tumor, pretreatment high LDH, rapid tumor growth, and large tumor border
Mott, 2005 (case 1) [134]	47	F	breast cancer	chemotherapy: fluorouracil/epiru bicin/cyclophosph amide, full dose	1 day after initiation	N/A	lethargy and lightheadedness	metastatic cancer
Mott, 2005 (case 2) [134]	44	F	breast cancer	chemotherapy: gemcitabine and cisplatin, full dose	1 day after initiation	N/A	nausea, dizziness, and decreased oral intake	metastatic cancer
Mott, 2005 (case 3) [134]	76	F	SCLC	chemotherapy: carboplatin and etoposide full dose	4 days after initiation	N/A	nausea and dehydration	metastatic cancer and high pretreatment LDH
Qian, 2009 [135]	44	M	primary retroperitoneal soft tissue sarcoma	chemotherapy: cisplatin 30 mg/m2 intravenously on days 1 through 4, doxorubicin 30 mg/m2 intravenously on days 1 and 3, dacarbazine 400 mg/m2 intravenously on days 1 through 3, full dose	3 days after initiation	N/A	drowsiness, chest tightness, palpitations, dyspnea, and oliguria	metastatic cancer and chemosensitivity
Yuan, 2017 [136]	43	M	GIST	targeted therapy: Imatinib 400mg, full dose	1 day after initiation	N/A	loss of consciousness	high tumor border and metastatic tumor
Lin, 2007 [137]	75	F	RCC	chemotherapy: gemcitabine monotherapy at a dosage of 1200 mg/m2 as a 30 minutes intravenous infusion, full dose	2 weeks after initiation	CKD	anorexia, fatigue, pedal edema, dyspnea, and anuria	metastatic cancer
Ling, 2012 [138]	40	M	pancreatic cancer	Chemotherapy: gemcitabine	2 days after initiation	N/A	nausea and vomiting	metastatic cancer
Sharma, 2006 [139]	63	M	HCC	chemotherapy: TACE (fluorouracil 1gm, Cisplatin 80mg, mitomycin 20mg and lipiodol 10ml), full dose	1 day after initiation	hepatitis B	fever, nausea, and oliguria	metastatic cancer
